# Triple-negative breast cancer: the reality in Chile and in Latin America

**DOI:** 10.3332/ecancer.2019.893

**Published:** 2019-01-22

**Authors:** Christian Caglevic, Jaime Anabalón, Cristian Soza, Elizabeth Milla, Fancy Gaete, Ana María Carrasco, Sergio Panay, Carlos Gallardo, Mauricio Mahave

**Affiliations:** 1 Medical Oncology Department, Clinica Alemana Santiago; Faculty of Medicine, Universidad del Desarrollo, Santiago 456, Chile; 2 Oncology Institute Arturo López Pérez Foundation, Santiago 878, Chile; 3 School of Biochemistry, Faculty of Science, San Sebastián University, Santiago 1457, Chile; 4 Oncoloop Foundation, Faculty of Medicine, Andrés Bello University, Santiago 890, Chile; 5 Hospital Santiago Oriente Dr Luis Tisné, Santiago 5150, Chile; 6 Oncology Institute Arturo López Pérez Foundation, Santiago 878, Chile

**Keywords:** breast cancer, triple-negative breast cancer

## Abstract

Breast cancer is the leading cause of cancer death among women worldwide. While triple-negative breast cancer is less common among various sub-types of breast cancer, it tends to affect younger women and is more aggressive, having a higher rate of early recurrence and mortality compared to other sub-types. We know about the association between triple-negative breast cancer and BRCA mutations, which are highly prevalent in founding populations of European origin, but the true prevalence of these mutations in Latin American populations is unknown. There is also very little information about the demographic and epidemiological aspects of triple-negative breast cancer in Latin America, which we will try to summarise in this article. In addition, we will try to provide a brief introduction to the most common recommendations for treating this histological class in Latin America.

## The epidemiology of breast cancer around the world and in Latin America

In women, breast cancer is the most common neoplasm and the second cause of death from cancer worldwide [1]. This pathology may present itself in various ways [2] and be related to multiple factors [3]. It is estimated that in 2012, 1.67 million new cases were diagnosed worldwide, corresponding to 25% of the total number of cancer cases [4]. Demographically speaking, both incidence and mortality rates vary according to the region, ethnic group and socioeconomic level analysed [1, 5, 6]. According to GLOBACAN 2012 estimates, in North America, the age-adjusted breast cancer incidence rate is 91.6 cases per 100,000 women and the age-standardised mortality rate is 14.8 cases per 100,000 women [4]. In the United States, despite the fact that breast cancer is still responsible for more than 40,000 deaths per year, the mortality rate has been decreasing continuously over the last 25 years [7]. In this way, it is estimated that for the decade of 2020–2030, breast cancer will continue to be the most common cancer, along with lung and prostate cancer, but its mortality rate will drop to lower than that of pancreas and liver cancers [8]. European countries follow a similar trend to that of the United States. The leading neoplasia in women in this region is also breast cancer, affecting more than one in 10 women, and accounting for 28.8% of all female cancers [9, 10].

Breast cancer has a high incidence rate in Latin America and is on the rise, so it is expected to double over the next decades [11, 12]. In fact, the incidence of this disease in Latin America and the Caribbean is 152,059 new cases (incidence of 42.7/100,000 adjusted by age), producing an age-adjusted mortality rate of 13 per 100,000 women [4]. The situation is made worse by the disparity in access to health services, diagnosis and detection, especially among ethnic minority communities and the lack of a good national cancer population registry in various countries [13]. In general, countries such as Mexico, Panama, Ecuador and Colombia have lower incidence rates than Central and Eastern Europe, similar to those of Asia. On the other hand, countries in the south of Latin America (Uruguay, Argentina and Chile) report higher incidence rates, similar to those of the United States and Europe [11], possibly due to greater access to healthcare and greater technological development, together with fewer people living outside the cities when compared to other countries in the region.

Incidence and mortality rates for this cancer have steadily increased over the last 25 years [14]. In Chile, for example, the increase in the crude incidence rate has, on average, been around 10.7% of the compound annual growth rate. This means an increase of 28.9 per 100,000 women in 2000 to 39.2 per 100,000 women in 2003 [15]. Similarly, in Brazil, the crude incidence rate has also increased from 22.9 per 100,000 women in 1988 to 68.2 per 100,000 women in 2003 [16].

The mortality rate in Latin American is variable, having decreased in some countries. For example, in Chile, the adjusted mortality rate increased from 1960 to the mid-1980s, but then between 1990 and 2010, there was a turning point, with a slight decrease [17, 18]. Demographic changes, ageing of the population and an increase in the incidence of this disease are factors that most impact the mortality rate in Latin America and the Caribbean. For example, it is estimated that, by 2030, the number of women over 60 will double and the World Health Organization estimates that by the same date, the number of deaths from breast cancer will increase from 43,208 to 73,542 cases in the region [19].

In Chile, detection and diagnosis programmes together with timely access to treatments have managed to reduce mortality rates despite socio-economic disparities and changes in lifestyles within the population. However, the lack of national cancer population registries makes it difficult to estimate accurately the mortality rate caused by breast cancer [15]. There are only three cancer population registries in Chile: two are located in the south of the country, in the Los Ríos Region and Bío-Bío Province, while the third is located in the Antofagasta Region in the north of Chile. Although numerically speaking, these records represent a low percentage of the population; they show how incidence and mortality rates in a country like Chile vary according to geography and various environmental contexts. For example, in the Antofagasta Region, the type of cancer with the highest incidence in women is non-melanoma skin cancer with a rate of 41.6 per 100,000 women, followed by breast cancer with a rate of 35.9 per 100,000 women. On the other hand, in the Los Ríos Region and Bío-Bío Province, breast cancer is the highest-occurring pathology with incidence rates of 40.7 and 35.3 per 100,000 women, respectively [17]. Breast cancer is not the leading cause of death in either of these regions. Instead, it is lung cancer (Antofagasta) and cancer of the gallbladder and bile ducts (Los Ríos Region and Bío-Bío Province) [20]. This shows the importance and usefulness of implementing national cancer registries in Latin American countries in order to establish strategies for detection and early diagnosis, in planning for access to appropriate treatments and prevention according to the major risk factors present. There is currently a huge effort being made in Chile to improve cancer registries, by seeking realistic ways to increase population sampling in order to have better statistics that may be useful for a better diagnosis and definition of public policies aimed at early diagnosis and timely treatment of cancer.

## Triple-negative breast cancer: molecular and demographic aspects, impact in Chile and Latin America

Following the original work of Perou, which showed the existence of intrinsic sub-types of breast cancer defined based on profiles of gene expression [21], a consensus classification based on immunohistochemical markers was established. Each of these groups has its own clinical behaviour with particular patterns of response to specific therapies in some cases.

In relation to the expression of oestrogen receptors, progesterone receptors and HER2 overexpression, four categories have been identified: Luminal A, Luminal B, HER2-amplified and Triple Negative, the latter of which includes tumours that do not express any of other three markers [22].

This group is heterogeneous, containing histological sub-types with especially good prognosis such as adenoid cystic carcinoma and varieties of greater aggressiveness such as metaplastic carcinoma. The basal-like sub-type defined by PAM50 is the most represented in the triple-negative category, where more than 90% of basal-likes are triple negative, and at the same time, basal-like is the most common sub-type within the triple negatives [23, 24]. Additionally, triple negatives have been subdivided into four more groups on the basis of gene expression, however, up to now, this sub-classification has had no real use in clinical practice [25].

According to various publications worldwide, between 15% and 20% of invasive breast tumours belong to the triple-negative group [26–28], with a higher occurrence among women under 40 years of age. The North American literature shows a higher incidence rate among patients of African descent, with a reported prevalence of up to 39%, followed by women of Hispanic origin. Population studies based on registries such as the California Cancer Registry and The Surveillance, Epidemiology, and End Results (SEER) Program of the National Cancer Institute (NCI) report a triple-negative disease prevalence of 12.5% that is strongly associated with patients under 40 years of age and of African descendent, in addition to low socio-economic levels and people with less access to healthcare [29–32].

In Latin America, breast cancer is the leading cause of death from tumours in young women. Triple-negative breast cancer is becoming a more common situation in young women, accounting for 20% of deaths due to breast cancer in the 45 years or younger age group, twice as much as the reported rate in developed countries. The reported prevalence of triple-negative disease in Latin America in young women ranges between 18% and 35%, with the highest rates being reported in countries such as Mexico and Peru [33].

Not much data on triple-negative disease has been published in Chile. A retrospective study in a university centre explored the situation of breast cancer at extreme ages (under 41 years of age and over 69) over a period of 16 years. 12% of patients in the registry were under 41 years of age, with Luminal B being the most frequent sub-type (43%), followed by Luminal A (33.2%) and triple-negative disease in third place (17.8%) [34]. Not yet published information from the Oncology Institute, Arturo López Pérez Foundation in Santiago, Chile reports that in 20 new cases of triple-negative breast cancer diagnosed during 2017, only 20% of patients were under the age of 40 and the average age of all patients was 49 years old, which shows an epidemiological discrepancy compared with known data from other populations of the same regional area. On the other hand, official data from the Luis Tisné Hospital in Santiago, Chile (flagship hospital for the diagnosis and treatment of breast cancer) reviewed for this work, showed that out of a total of 285 biopsies performed on patients with breast cancer between June 2014 and May 2015, only 8.7% corresponded to the triple-negative sub-type, thus showing a much lower prevalence of this sub-type of breast cancer in a representative sample of Chilean women with breast cancer when compared with other groups in Latin America.

Unfortunately, there is a regional lack in our continent in terms of having real epidemiological knowledge of many oncological pathologies, and in particular, the epidemiology of triple-negative breast cancer. Neither is there any certainty of the magnitude of the problem nor of the differences among countries and among regions within the same country, considering that most of the published data about breast cancer involve the total number of patients independent of the histological sub-types. In the understanding of the most aggressive natural history of triple-negative breast cancer, it is necessary to consider the report of this sub-type of breast cancer within the various health systems of Latin America, given that it may have potential implications in projecting health policies aimed at attacking this disease more quickly and more aggressively in consideration of its high mortality rate and poor prognosis.

## Clinical presentation and natural history

Compared with Luminal A and Luminal B type tumours, the triple-negative sub-type or variety tends to occur with a larger size, a higher histologic degree and a faster growth. In fact, they can often be investigated as ‘interval tumours’ during screening programmes. In relation to a possible greater lymph-node involvement, there is no clearly demonstrated association so far. Its frequent metastatic presentation is characterised by a greater visceral involvement, particularly in the central nervous system and lungs, and a lower frequency of bone involvement [35].

These tumours are generally associated with a worse prognosis in relation to luminal tumours, with a less than 80% chance of surviving 5 years, on average, compared with non-triple-negative tumours, where the chance of surviving 5 years exceeds 90%, according to U.S. data. There are limitations in the understanding of the underlying biology, as well as the lack of specific therapeutic agents for its treatment. Its recurrences are early and systemic, with a significantly higher risk of death during the first 5 years following diagnosis. Up to 50% of patients diagnosed with non-metastatic triple-negative breast cancer will become ill again and 37% will die from the disease during the first 5 years after surgery [36, 37]. What was shown is that it has the highest recurrence-free survival rate in patients who underwent neoadjuvant chemotherapy and who managed a pathologic complete response compared to women who did not achieve this goal (HR 0.24 (95% CI 0.18–0.33)) [38]. The use of platinum in combination with anthracyclines and taxanes demonstrated a statistically better pathologic complete response (53%) compared with patients who did not use carboplatin (39%) with triple-negative breast cancer stages II and III [39], behaviour that in the end may result in a greater hope for recurrence-free survival and, indirectly, the overall survival of these patients. The benefit of using carboplatin-based treatments shows even better results in a pathologic complete response in those groups of patients who are carriers of BRCA mutations [40]. In the presence of metastasis, the median survival is between 9 and 12 months, compared to 2–4 years observed in other sub-types of breast cancer. [41]

Patients with metastatic disease have a short progression-free survival expectancy following the failure of the first-line chemotherapy with an average of no longer than 4 months, indicating the great need to develop drugs to treat triple-negative breast cancer**.** With the exception of Olaparib for the treatment of tumours with BRCA germline mutations [42], there are currently no other approved therapies for this group, leaving cytotoxic chemotherapy as a pillar of systemic treatment. On the other hand, the actual prevalence of BRCA mutations is not well known among the various towns and regions of Latin America. There are varying results in different populations within the same country and in populations selected by genetic risk criteria [43, 44].

Various recommendation guidelines for Latin American regarding the management of early and metastatic breast cancer recognise triple-negative breast cancer as a high-risk variety, however, they do not suggest the use of different therapies from those already commonly used for other sub-types (excluding targeted therapies for Luminal HER2), such as treatments based on anthracyclines and taxanes. Triple-negative breast cancer has higher response rates to neoadjuvant chemotherapy in comparison with other sub-types of breast cancer and is known as the triple-negative breast cancer paradox [45]. Advantages of neoadjuvancy include minimising the size of the tumour to allow breast-conserving surgery in selected cases, reducing options for extensive axillary dissection, making an inoperable tumour resectable and allowing an *in vivo* evaluation of the sensitivity of the tumour to chemotherapy, thereby decreasing the possibilities of metastasis. There is a shortage of public policies and management guidelines on early disease that include studies on BRCA mutations and the use of platinum duplicates in neoadjuvant therapies. In a meta-analysis involving 11,955 patients undergoing neoadjuvant chemotherapy for breast cancer, the pathologic complete response showed a significant correlation with disease-free survival and overall survival [38]. Patients who achieved a pathologic complete response had significantly better results. Recurrence-free survival with an HR of 0.24 and overall survival with an HR of 0.16 indicate that pathologic complete response has a prognostic value in triple-negative breast cancer. The most used adjuvant systemic treatment in Latin America, where neoadjuvancy was not used previously, includes the sequential combination of chemotherapy based on anthracyclines followed by taxanes. Similar confrontation occurs in metastatic disease where, since hormonal therapies cannot be used, the range of options is limited today to systemic therapies which include anthracyclines, taxanes, platinum-based duplicates (e.g. a combination of carboplatin and gemcitabine) and capecitabine, ixabepilone, among other options.

In addition to available treatments, considering the poor prognosis of triple-negative breast cancer and the strong aggressiveness of metastatic disease added to the ignorance of the real incidence and prevalence of triple-negative disease in our populations, as well as the urgent need for new treatment options, it makes sense to involve patients in clinical studies [46] and give patients the option of receiving new therapeutic options that can potentially affect a better prognosis and survival rate (Table 1).

## Conclusions

Breast cancer is the leading cause of death in women around the world. Among the sub-types classified by Perou, triple-negative breast cancer has biological characteristics that make it a sub-type with a more aggressive presentation at earlier ages, a very high risk of mortality and/or a quick recurrence rate and, at the same time, high and early failure of systemic therapy in metastatic disease.

In Israel and Europe, triple-negative breast cancer, like ovarian cancer, has been widely associated with populations having a high prevalence of BRCA gene mutations (BRCA 1 and BRCA 2); a situation occurring in various populations worldwide in women who are carriers of these mutations. In Latin America where the known presence of BRCA mutations is much lower than in the other regions mentioned, the prevalence of triple-negative breast cancer ranges between 8% and 35% according to different reports. However, the prevalence of this problem is not known for certain because little data has been reported on the various sub-types in the country, and we are limited to information published in reports on regional experiences and series, including information presented at scientific events but not published.

This disinformation must be improved in order to consider health policies that focus on the triple-negative disease; a sub-type of breast cancer with its own characteristics such as its aggressiveness, its occurrence mainly in young women, its high rate of metastasis and mortality when compared with other histological sub-types, all of which convert this sub-type into a unique variety with a worse prognosis.

In locoregional disease, neoadjuvant chemotherapy associated with a pathologic complete response has been shown to have an impact on the survival of these patients. For this reason, this method, often discarded in Latin America for practical reasons such as a lack of rapid access to cancer centres, a lack of knowledge about the natural history of triple-negative breast cancer and the lack of multidisciplinary cancer management in some areas, should standardise the use of neoadjuvant chemotherapy in all women in Latin America with triple-negative non-metastatic breast cancer, ideally with combinations containing carboplatin or cisplatin. Unfortunately, to date, the prognosis of the metastatic disease is poor and the option of managing the disease is limited to various palliative chemotherapy options. However, the possibility of including these patients in clinical studies, even women with the locoregional disease, would be highly recommended.

## Conflicts of interest

This publication has not been funded by any industry or by any institution. None of the authors have received payment of any kind for this publication. Christian Caglevic declares the following conflicts of interest:

- Speaker: MSD, GSK, Bayer, BMS, Boehringer Ingelheim, Tecnofarma, Roche
- Principal Investigator: MSD, GSK, Bayer, Boehringer Ingelheim, Astellas, Roche, Astra Zeneca, BMS, Novartis
- Advisory and Consulting: MSD, BMS, Bayer, Astra Zeneca, Boehringer Ingelheim, Lilly, Tecnofarma
- Sponsored Educational Programme: Boehringer Ingelheim, Tecnofarma, BMS, MSD

## Figures and Tables

**Table 1.** Clinical studies in development for patients with triple-negative breast cancer.

Category	Details of the Study	Phase	NCT
**Inhibitors checkpoint**	Nab-paclitaxel ± atezolizumab (MPDL3280A) in previously untreated patients *(IMpassion130)*	**III**	**NCT02425891**
	Study of Pembrolizumab (MK-3475) plus chemotherapy versus chemotherapy plus placebo for inoperable, locally recurring or previously untreated metastatic triple-negative breast cancer (MK-3475-355 / *KEYNOTE-355*)	**III**	**NCT02819518**
**PARP inhibitors**	A Phase 3, open-label, randomized parallel, 2-arm, multi-centre study Of Talazoparib (Bmn 673) versus physician’s choice in germline BRCA mutation subjects with locally advanced and/or metastatic breast cancer, who have received prior chemotherapy regimens for metastatic disease	**III**	**NCT01945775**
**Antiandrogen therapy**	Bicalutamide versus first-line chemotherapy in androgen receptor positive triple-negative metastatic breast cancer	**III**	**NCT03055312**
**PI3K-AKT-mTOR inhibitors**	Paclitaxel + AZD5363 in first line metastatic (PAKT)	**II**	**NCT02423603**
	BYL719 monotherapy in metastatic breast cancer (second line)	**II**	**NCT02506556**
**Conjugated antibodies**	Randomised, sacituzumab govitecan (IMMU-132) versus treatment of the doctor’s choice in patients with triple-negative metastatic breast cancer who have received at least two lines of previous treatments	**III**	**NCT02574455**

## References

[1] Hiatt RA, Brody JG (2018). Environmental determinants of breast cancer. Annu Rev Public Health.

[2] Kohler BA, Sherman RL, Howlader N (2015). Annual report to the nation on the status of cancer, 1975-2011, featuring incidence of breast cancer subtypes by race/ethnicity, poverty, and state. J Natl Cancer Inst.

[3] Hiatt RA, Porco TC, Liu F (2014). A multilevel model of postmenopausal breast cancer incidence. Cancer Epidemiol Biomarkers Prev.

[4] Ferlay J, Soerjomataram I, Ervik M (2012). GLOBOCAN 2012 v1.0, Cancer incidence and mortality worldwide: IARC cancerbase No. 11.

[5] Smith BD, Smith GL, Hurria A (2009). Future of cancer incidence in the United States: burdens upon an aging, changing nation. J Clin Oncol.

[6] DeSantis CE, Ma J, Goding Sauer A (2017). Breast cancer statistics, 2017, racial disparity in mortality by state. CA Cancer J Clin.

[7] Howlader N, Noone AM, Krapcho M (2016). SEER Cancer statistics review, 1975-2013.

[8] Rahib L, Smith BD, Aizenberg R (2014). Projecting cancer incidence and deaths to 2030: the unexpected burden of thyroid, liver, and pancreas cancers in the United States. Cancer Res.

[9] Lundqvist A, Andersson E, Ahlberg I (2016). Socioeconomic inequalities in breast cancer incidence and mortality in Europe-a systematic review and meta-analysis. Eur J Public Health.

[10] Ferlay J, Steliarova-Foucher E, Lortet-Tieulent J (2013). Cancer incidence and mortality patterns in Europe: estimates for 40 countries in 2012. Eur J Cancer.

[11] Justo N, Wilking N, Jönsson B (2013). A review of breast cancer care and outcomes in Latin America. Oncologist.

[12] Anderson BO, Yip CH, Smith RA (2008). Guideline implementation for breast healthcare in low-income and middle-income countries: overview of the Breast Health Global Initiative Global Summit 2007. Cancer.

[13] Goss PE, Lee BL, Badovinac-Crnjevic T (2013). Planning cancer control in Latin America and the Caribbean. Lancet Oncol.

[14] Lozano-Ascencio R, Gómez-Dantés H, Lewis S (2009). Breast cancer trends in Latin America and the Caribbean. Salud Publica Mex.

[15] MTS P (2006). Situación epidemiológica del cáncer de mama en Chile 1994–2003. Rev Med ClinCondes.

[16] Freitas R, Freitas NM, Curado MP (2010). Incidence trend for breast cancer among young women in Goiania, Brazil. Sao Paulo Med J.

[17] Laura Itriago G, Nicolas Silva I, Giovanna Cortes F (2013). Cáncer en chile y el mundo: unamirada epidemiológica, presente y futuro. Rev Med Clin Condes.

[18] Carioli G, La Vecchia C, Bertuccio P (2017). Cancer mortality predictions for 2017 in Latin America. Ann Oncol.

[19] World Health Organization (WHO) (2008). The global burden of disease: 2004 summary tables. October 2008 update.

[20] Chile MDSD (2012). Primer informe de registro poblacional de cancer en chile. Quinquenio 2003-2007. Unidad de vigilancia de enfermedades no transmisibles y estudios departamento de epidemiología división planificación sanitaria subsecretaría de salud pública.

[21] Perou CM, Sørlie T, Eisen MB (2000). Molecular portraits of human breast tumours. Nature.

[22] Curigliano G, Burstein HJ, Winer EP (2017). De-escalating and escalating treatments for early-stage breast cancer: the St. Gallen International Expert Consensus Conference on the Primary Therapy of Early Breast Cancer 2017. Ann Oncol.

[23] Cheang MC, Martin M, Nielsen TO (2015). Defining breast cancer intrinsic subtypes by quantitative receptor expression. Oncology.

[24] Prat A, Adamo B, Cheang MC (2013). Molecular characterization of basal-like and non-basal-like triple-negative breast cancer. Oncology.

[25] Lehmann BD, Jovanović B, Chen X (2016). Refinement of triple-negative breast cancer molecular subtypes: implications for neoadjuvant chemotherapy selection. PLoS One.

[26] Boyle P (2012). Triple-negative breast cancer: epidemiological considerations and recommendations. Ann Oncol.

[27] Foulkes WD, Smith IE, Reis-Filho JS (2010). Triple-negative breast cancer. N Engl J Med.

[28] Dent R, Trudeau M, Pritchard KI (2007). Triple-negative breast cancer: clinical features and patterns of recurrence. Clin Cancer Res.

[29] Carey LA, Perou CM, Livasy CA (2006). Race, breast cancer subtypes, and survival in the carolina breast cancer study. JAMA.

[30] Bauer KR, Brown M, Cress RD (2007). Descriptive analysis of estrogen receptor (ER)-negative, progesterone receptor (PR)-negative, and HER2-negative invasive breast cancer, the so-called triple-negative phenotype: a population-based study from the california cancer registry. Cancer.

[31] Harris LN, Broadwater G, Lin NU (2006). Molecular subtypes of breast cancer in relation to paclitaxel response and outcomes in women with metastatic disease: results from CALGB 9342. Breast Cancer Res.

[32] Howlader N, Altekruse SF, Li CI (2014). US incidence of breast cancer subtypes defined by joint hormone receptor and HER2 status. J Natl Cancer Inst.

[33] Villarreal-Garza C, Aguila C, Magallanes-Hoyos MC (2013). Breast cancer in young women in Latin America: an unmet, growing burden. Oncologist.

[34] Acevedo F, Camus M, Sanchez C (2015). Breast cancer at extreme ages–a comparative analysis in Chile. Asian Pac J Cancer Prev.

[35] Bianchini G, Balko JM, Mayer IA (2016). Triple-negative breast cancer: challenges and opportunities of a heterogeneous disease. Nat Rev Clin Oncol.

[36] Liedtke C, Mazouni C, Hess KR (2008). Response to neoadjuvant therapy and long–term survival in patients with triple-negative breast cancer. J Clin Oncol.

[37] Guarneri V, Broglio K, Kau SW (2006). Prognostic value of pathologic complete response after primary chemotherapy in relation to hormone receptor status and other factors. J Clin Oncol.

[38] Cortazar P, Zhang L, Untch M (2014). Pathological complete response and long–term clinical benefit in breast cancer: the CTNeoBC pooled analysis. Lancet.

[39] von Minckwitz G, Schneeweiss A, Loibl S (2014). Neoadjuvant carboplatin in patients with triple-negative and HER2-positive early breast cancer (GeparSixto; GBG 66): a randomised phase 2 trial. Lancet Oncol.

[40] Hahnen E, Lederer B, Hauke J (2017). Germline mutation status, pathological complete response, and disease-free survival in triple-negative breast cancer: secondary analysis of the geparSixto randomized clinical trial. JAMA Oncol.

[41] Denkert C, Liedtke C, Tutt A (2017). Molecular alterations in triple-negative breast cancer-the road to new treatment strategies. Lancet.

[42] Robson M, Im S, Senkus E (2017). Olaparib for metastatic breast cancer in patients with a germline BRCA mutation. NEJM.

[43] Sanabria M, Muñoz G, Vargas C (2009). Análisis de las mutaciones más frecuentes del gen BRCA1 en mujeres con cáncer de mama en Bucaramanga, Colombia. Revista Biomédica.

[44] Arias-Blanco F, Ospino-Durán J, Restrepo-Fernández E (2015). Prevalencia de mutación y de variantes de secuencia para los genes BRCA1 y BRCA2 en una muestra de mujeres colombianas con sospecha de síndrome de cáncer de mama hereditario: serie de casos. Revista Colombiana de Obstetricia y Ginecología.

[45] Carey LA, Dees EC, Sawyer L (2007). The triple negative paradox: primary tumor chemosensitivity of breast cancer subtypes. Clin Cancer Res.

[46] Rolfo C, Caglevic C, Bretel D (2016). Cancer clinical research in Latin America: current situation and opportunities. Expert opinion from the first ESMO workshop on clinical trials, Lima, 2015. ESMO Open.

